# Mindfulness training promotes upward spirals of positive affect and cognition: multilevel and autoregressive latent trajectory modeling analyses

**DOI:** 10.3389/fpsyg.2015.00015

**Published:** 2015-02-02

**Authors:** Eric L. Garland, Nicole Geschwind, Frenk Peeters, Marieke Wichers

**Affiliations:** ^1^College Of Social Work, University of Utah, Salt Lake CityUT, USA; ^2^Integrative Medicine – Supportive Oncology, Huntsman Cancer Institute, University of UtahSalt Lake City, UT, USA; ^3^Maastricht UniversityMaastricht, Netherlands; ^4^University of GroningenGroningen, Netherlands

**Keywords:** mindfulness training, positive emotion, broaden-and-build, emotion regulation, latent growth curve analysis

## Abstract

Recent theory suggests that positive psychological processes integral to health may be energized through the self-reinforcing dynamics of an upward spiral to counter emotion dysregulation. The present study examined positive emotion–cognition interactions among individuals in partial remission from depression who had been randomly assigned to treatment with mindfulness-based cognitive therapy (MBCT; *n* = 64) or a waitlist control condition (*n* = 66). We hypothesized that MBCT stimulates upward spirals by increasing positive affect and positive cognition. Experience sampling assessed changes in affect and cognition during 6 days before and after treatment, which were analyzed with a series of multilevel and autoregressive latent trajectory models. Findings suggest that MBCT was associated with significant increases in trait positive affect and momentary positive cognition, which were preserved through autoregressive and cross-lagged effects driven by global emotional tone. Findings suggest that daily positive affect and cognition are maintained by an upward spiral that might be promoted by mindfulness training.

## INTRODUCTION

The dynamic nature of human experience emerges from the continual demand to adapt to changing and challenging life circumstances; it is out of this process of adaptation that emotions arise (Lazarus, 1991). In turn, emotions are thought to flow from appraisal of the meaning of one’s internal state and current environmental context. When life circumstances are appraised to be benign, beneficial, or rewarding, positive affect results (Lazarus and Folkman, 1984). In complementary fashion, when individuals experience positive emotion, they are more likely to notice the pleasant, beautiful, or rewarding aspects of their lives (Tamir and Robinson, 2007), and are more likely to adopt a positive attitude to self or others when making evaluative judgments (Clore and Palmer, 2009). Thus, positive cognitive processes and positive affect appear to be tightly linked. In previous theoretical work, we (Garland et al., 2010) have proposed that positive psychological processes energize and maintain one another through the self-reinforcing dynamics of a positive feedback loop, which we and others (Fredrickson and Joiner, 2002; Burns et al., 2008; Kok and Fredrickson, 2011) have termed an *upward spiral*. Yet, at present, few empirical studies have elucidated the dynamics of positive emotion–cognition interactions.

In contrast, substantially more is known about how negative affect influences cognition, and in turn, how negatively biased cognitive processes amplify and further entrench negative affect (Mathews and MacLeod, 2005). Such negatively valenced emotion–cognition interactions are particularly evident in depression. Depressed individuals preferentially attend to negative stimuli (e.g., sad faces; Gotlib et al., 2004), ruminate on negative beliefs about the self, world, and future (Nolen-Hoeksema, 2000), resolve ambiguous stimuli negatively (Mogg et al., 2006), and make negative appraisals of the valence of their own thoughts (Freeston et al., 1995) – i.e., *negative thought appraisals* (Purdon et al., 2005). These negative cognitive processes may explain why depressed individuals have fewer positive thoughts than non-depressed people (Ingram et al., 1990). Thus biased information processing in depression decreases the pleasantness of thoughts and produces feelings of dysphoria, processes which may be underpinned by alterations to functional and structural neural architecture that increase the influence of subcortical brain regions while impairing top–down, prefrontal cognitive control (Disner et al., 2011). As a possible result of functional imbalances in cortical–subcortical networks, depressed individuals are unable to sustain activations in brain regions that subserve the experience of reward (e.g., nucleus accumbens), thus accounting in part for their lower levels of positive affect (Heller et al., 2009). Indeed, a number of studies demonstrate that depressed individuals experience less daily positive affect (Peeters et al., 2006). Deprived of the ability to sustain and increase positive affect over time, depressed persons may remain mired in a downward spiral of negativity that perpetuates emotion dysregulation and prolongs depression (Garland et al., 2010; Wichers et al., 2010).

As such, positive affect may be central to countering the dysfunctional cognition–emotion interactions in depression. According to Fredrickson’s (1998, 2004) broaden-and-build theory, positive emotions undo the psychophysiological consequences of negative emotions (Fredrickson and Levenson, 1998), and expand the scope of cognition to allow individuals to access a wider-than-usual range of percepts, associations, ideas, and action urges (Fredrickson and Branigan, 2005). In addition to broadening the scope of cognition, positive affect may also increase the frequency and intensity of positively valenced cognitions (i.e., *positive cognitions*, see Macleod and Moore, 2000), by biasing attention (Wadlinger and Isaacowitz, 2010) and memory recall (Roberts-Wolfe et al., 2012) toward positive information, promoting positive interpretations of ambiguous situations (Mathews, 2012), and assigning positive value to objects or thoughts during cognitive appraisal (Clore and Huntsinger, 2007). Through these mechanisms, positive emotions foster positive cognitions that promote novel and exploratory sequences of behavior (Cohn et al., 2009), which creates more opportunities to encounter rewarding life experiences – the “fuel” for increased positive appraisals and emotions in the future. In these ways, upward spirals of positive emotion and cognition may exert a countervailing force of the dysphoric and anhedonic states characteristic of depression.

Plausibly, interventions and experiences that engender positive affective and cognitive states may amplify upward spirals above and beyond preexisting trait-like deficits in positive affect and cognition. One form of mental training that may stimulate upward spirals of positive emotion is mindfulness meditation. The practice of mindfulness meditation involves repeated placement of attention onto an object (such the sensory experience of breathing, walking, or other common activities) while accepting and letting go of distracting thoughts and emotions. This practice is thought to engender a specific metacognitive state: a non-reactive, non-evaluative monitoring of moment-by-moment cognition, emotion, perception, and sensation without fixation on thoughts of past and future (Garland, 2007; Lutz et al., 2008). Ultimately, recurrent practice of engaging the state of mindfulness is thought to develop dispositional mindfulness, the propensity to experience and express mindful attitudes in everyday life (Baer et al., 2006).

As investigators increasingly incorporate measures of positive emotion into their research protocols, more studies have identified effects of mindfulness training on positive affective processes. Community samples undergoing mindfulness training have evidenced significant increases in positive affect (Nyklíček and Kuijpers, 2008; Orzech et al., 2009). Mindfulness training has also been shown to influence positive affect among clinical samples, such as patients with comorbid recurrent depression and rheumatoid arthritis (Zautra et al., 2008). Notably, a RCT of adults with residual depressive symptoms found that mindfulness-based cognitive therapy (MBCT; Segal et al., 2002) increased positive affect and the sense of reward from savoring pleasant daily life activities (Geschwind et al., 2011). Mindfulness training was also found to enhance reward responsiveness in a RCT of opioid misusing chronic pain patients as evidenced by increased cardiac-autonomic (Garland et al., 2014a) and electrocortical responses (Garland et al., 2014b) to positive affective stimuli.

Similarly, mindfulness training may influence positive cognitive processes. For instance, mindfulness training was associated with significantly improved memory for positive information among a community sample (Roberts-Wolfe et al., 2012). Beyond its effects on memory, mindfulness training may also promote positive reappraisal (Garland et al., 2009), the capacity to reappraise negative or stressful life events as benign, beneficial, or meaningful (Lazarus and Folkman, 1984). Indeed, increases in dispositional mindfulness over the course of a mindfulness training program were reciprocally linked with increases in positive reappraisal, and that the stress-reductive effects of increases in dispositional mindfulness were partially mediated by increases in positive reappraisal (Garland et al., 2011). Another study found that relative to individuals in two control conditions, those who had been treated with MBCT evidenced significantly greater positive reappraisal ability during an experimental sad mood induction (Troy et al., 2012). And, a recent RCT found that a mindfulness-oriented intervention led to significantly greater increases in positive reappraisal than a support group control (Garland et al., 2014c).

While these studies are promising, little is known about how mindfulness training might modulate interactions between positive affect and cognition over time. Recently, Garland and Fredrickson (2013) proposed a novel theoretical model to explicate how mindfulness might stimulate upward spirals of positive affect and cognition. In brief, the practice of mindfulness is hypothesized to evoke a metacognitive state of broadened awareness with downstream effects on affect and cognition. Insofar as positive emotions may broaden cognition (Fredrickson and Branigan, 2005), the broadened scope of attention induced by mindfulness practice may in turn induce positive emotions (Garland et al., 2010). The consequent positive affective state may then bias and tune attention (Friedman and Förster, 2010) toward an expanded set of contextual information from which positive appraisals of external and internal stimuli (mental contents: e.g., thoughts) can be generated. When the consequent positive cognitions and positively valenced emotional states become the new target of mindful awareness, this amplifies positive emotion and increases attentional tuning toward mood-congruent contextual features that support further positive appraisals and a deepening of positive emotion. Thus, when paid mindful attention, momentary experiences of positive affect and cognition may interact synergistically in an autoregressive and cross-lagged fashion and thereby energize the daily maintenance and gradual development of well-being and resilience. Through the prospective cyclical relation of the upward spiral dynamic, time-specific elevations in positive affect stimulated by mindfulness practice (above and beyond an individual’s average level of positive affect) are presumed to predict subsequent time-specific elevations in positive cognitions (above and beyond an individual’s average level of positive cognitions), and vice versa. In this sense, mindfulness provides the perturbation or shock that reverberates through the cognitive-emotional system, resulting in momentary psychological elevations that feed back into the system to tip one’s dispositional affective style (Davidson, 2004) toward a more durable positivity.

In view of this theoretical model, the purpose of the present study is to elucidate the temporal dynamics of positive emotion–cognition interactions among a sample of individuals in partial remission from depression who had been randomly assigned to treatment with MBCT or a waitlist control condition. Data from this RCT were previously analyzed by Geschwind et al. (2011) to test the effects of MBCT on reward experience. The experience sampling method (ESM) was used to measure dynamic changes in affect and cognition to test a number of hypotheses that were distinct from those tested in this earlier investigation. Our primary aim was to test the hypothesis that MBCT will significantly increase momentary positive cognitions relative to a waitlist control group. Although positive cognition might be evidenced by positive attentional bias, positive memory bias, positive interpretation bias, etc., in this study, positive cognition was operationalized as appraising one’s current thoughts as positively valenced – i.e., positive thought appraisal (c.f., Purdon and Clark, 2001). Our secondary aim was to explore how MBCT might influence the temporal dynamics of positive affect and cognition in this sample and provide a partial test of the upward spiral model. In that regard, we had several hypotheses, which, if taken together and supported by the data, comprise evidence for the upward spiral model: (a) elevations in positive affect on a given day will predict elevations in positive affect on the following day, above and beyond any trait-like propensity toward positive affect (*autoregressive component of positive affect*); (b) elevations in positive cognition on a given day will predict elevations in positive cognition on the following day, above and beyond any trait-like propensity toward positive cognition (*autoregressive component of positive cognition*); (c) elevations in positive affect on a given day will predict elevations in positive cognition on the following day, and vice versa (*cross-lagged relations between positive affect and cognition*); (d) MBCT will stimulate an upward spiral dynamic in these emotion–cognition interactions by strengthening the cross-lagged relationship between positive affect and cognition and providing the initial elevation in these variables that will then be carried forward in an autoregressive fashion.

## MATERIALS AND METHODS

### PARTICIPANT CHARACTERISTICS

Adults diagnosed with at least one major depressive episode who had residual depressive symptoms were recruited from outpatient mental health treatment facilities in Maastricht (the Netherlands) and through flyers posted in public spaces. The presence of residual depressive symptoms was operationalized as scoring seven or higher on the 17-item Hamilton Depression Rating Scale (HDRS; Hamilton, 1960) at the time of screening. Potential participants were excluded if they fulfilled criteria for a current depressive episode, schizophrenia, or psychotic episodes in the past year, and if they had recent (past 4 weeks) or upcoming changes in ongoing psychological or pharmacological treatment. The average ages of participants randomly assigned to the MBCT and waitlist control conditions were 44.6 (SD = 9.7) and 43.2 (SD = 9.5), respectively. The majority of participants were female (MBCT: 79%; control group: 73%). A substantial number of participants had had three or more previous depressive episodes (MBCT: 44%; control group: 45%). Other relevant sociodemographic and clinical characteristics are reported in Geschwind et al. (2011).

### SAMPLING PROCEDURES

**FIGURE 1:**
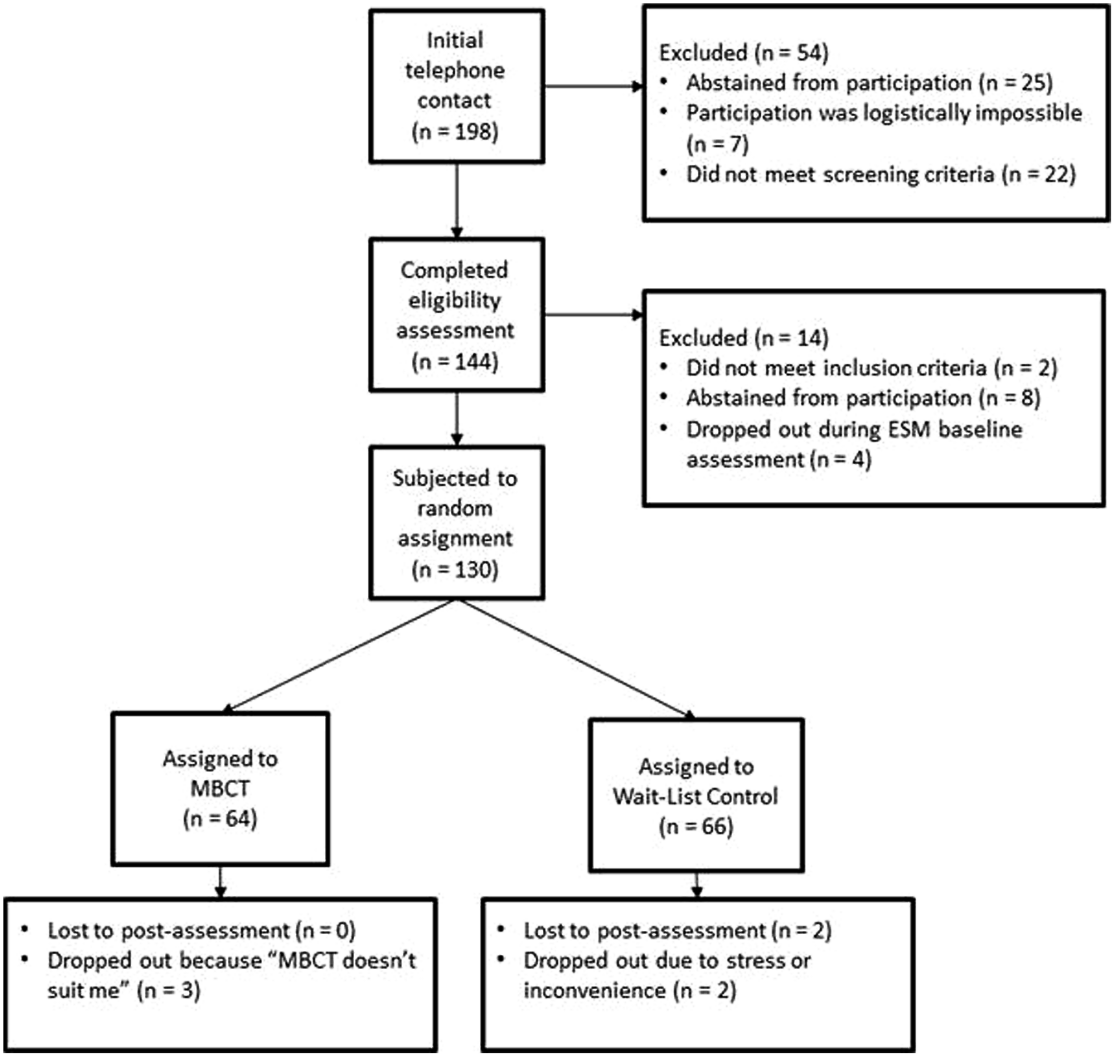
**Study flow diagram**.

Potential participants participated in an initial phone screen to establish inclusion and exclusion criteria. During a second screening, participants were administered the Structured Clinical Interview for *DSM IV*–Axis I (First et al., 2002) and the 17-item HDRS by trained psychologists. Eligible participants were trained in using the ESM procedure, and then completed a pre-treatment assessment consisting of 6 days of experience sampling in their own environment (see Experience Sampling Method). After the baseline assessment, participants were randomized to either 8 weeks of MBCT or 8 weeks of a waitlist control condition (allocation ratio 1:1). In light of previous studies that suggest a greater benefit for those with three or more previous episodes (Teasdale et al., 2000; Ma and Teasdale, 2004), randomization to treatment condition was stratified according to number of depressive episodes (two or less vs. three or more). An independent investigator not involved in the project generated the random allocation sequence via computer in blocks of five. After completion of all baseline assessments, the researcher allocated participants to their treatment condition based on their randomization code in a sealed envelope that was opened in order of sequence. No masking of treatment condition took place. Following MBCT or the waiting period, participants again took part in 6 days of experience sampling. For their completion of the research, participants were compensated with gift vouchers worth 50 Euros. After the post-intervention assessment, individuals in the waitlist condition had the opportunity to participate in MBCT. Study procedures were approved by the Medical Ethics Committee of Maastricht University Medical Centre. The study flow diagram is depicted in **Figure 1**.

### MBCT INTERVENTION

Mindfulness-based cognitive therapy training sessions closely followed the protocol outlined in Segal et al. (2002). MBCT sessions consisted of 8 weekly, 2.5 h and were run in groups of 10–15 participants (thus occasionally larger than the usual 10–12 participants per group). The time frame of assessments for control participants was matched to those of MBCT participants. MBCT sessions included instructor-led mindfulness meditation, experiential exercises, and group discussion. Participants received CDs with guided mindfulness exercises and were assigned 30–60 min of homework practice a day. MBCT was delivered by experienced trainers in a center that regularly offered mindfulness training. All trainers were supervised by an experienced health care professional who had been trained in MBCT by its co-developers, Teasdale and Williams. The mean number of MBCT sessions attended was 7.2 (SD = 1.5).

### EXPERIENCE SAMPLING METHOD

Experience sampling method is a momentary assessment method that assesses participants in their daily living environment. As such, this method provides repeated, in-the moment assessments of psychological experience in a prospective and ecologically valid manner (Csikszentmihalyi and Larson, 1987; Peeters et al., 2003). ESM offers several advantages over retrospective questionnaires and interviews, including increased ecological validity, minimized retrospective bias, and improved reliability (Csikszentmihalyi and Larson, 1987). In the current study, ESM was conducted via self-assessment forms collated in a booklet for each day whose completion was prompted by a wristwatch programmed to emit a signal (“beep”) at an unpredictable moment in each of ten 90-min time blocks between 7:30 a.m. and 10:30 p.m. ESM was conducted for six consecutive days for each study period (pre-intervention and post-intervention), resulting in a maximum of 60 beeps per study period. All self-assessments were rated on 7-point Likert scales. Trained research assistants explained the ESM procedure to the participants during an initial briefing session, and a practice form was completed to confirm that participants understood the 7-point Likert scale. To minimize memory distortion, participants were instructed to complete their ESM reports immediately after each beep, and to record the time at which they completed the form. All reports not filled in within 15 min after the actual beep were excluded from this analysis, due to decreased reliability and validity for reports completed after this interval (Delespaul, 1995). Data from a circumscribed subset of the complete ESM measurement package are reported in the present study, as detailed below.

#### Measurement of positive affect

At each beep, several ESM mood adjectives were assessed on 7-point Likert scales ranging from 1 (*not at all*) to 7 (*very*). Consistent with previous work (Myin-Germeys et al., 2001; Wichers et al., 2010), principal component factor analysis with oblique rotation was used to generate a factor representing PA. The mood adjectives “happy,” “satisfied,” “strong,” “enthusiastic,” “curious,” “animated,” and “inspired” loaded on the PA factor (α = 0.89). One mood item (“I feel relaxed”) was not included in the PA factor due to low factor loadings (<0.6). Mean levels of PA were then computed per participant for each beep moment.

#### Measurement of positive cognition

At each beep, participants were also asked the open-ended question “What am I thinking right now?” Subjects were encouraged to write down the first thought on their mind (this qualitative data will not be reported in the present manuscript). They were explicitly discouraged to write down chains of thoughts or multiple thoughts. Subsequently, to assess for the presence of positive cognition (i.e., positive thought appraisal), participants were asked to rate to what extent they agreed with the phrase “This is a pleasant thought” on a 7-point Likert scale ranging from 1 (*not at all*) to 7 (*very*) at each beep. If the thought was wholly unpleasant, they would rate that thought as a 0 on the pleasantness scale. Purdon and Clark (2001) and Purdon et al. (2005) assessed positive vs. negative valence of cognition employing a similar strategy of asking participants to rate the pleasantness of their thoughts.

### DATA ANALYTIC PLAN

Our primary study hypothesis that MBCT would increase momentary positive cognition was tested with a multilevel linear regression model (Singer and Willett, 2003). For this analysis, ESM data were analyzed within a hierarchical structure. Multiple observations (Level 1) are clustered within participants (Level 2). Multilevel linear regression using the linear mixed modeling command in SPSS 20.0 (IBM Statistics, 2012) examined the effects of MBCT on change in momentary positive cognition. The two-way interaction between time (baseline vs. post-assessment) and treatment group (control vs. MBCT) was the parameter of interest. For this analysis, we allowed intercepts to vary randomly. Because we assumed the presence of autoregressive effects from one momentary cognitive state to the next, a first order autoregressive term (that is, AR[1]) was included in the repeated measures model.

Secondary study hypotheses were tested with a series of autoregressive latent trajectory (ALT) growth models (Bollen and Curran, 2004) following the analytic methodology employed by Rodebaugh et al. (2002). Bollen and Curran’s (2004) ALT modeling strategy allows us to simultaneously estimate the time-specific autoregressive and cross-lagged effects of daily positive affect and cognition on succeeding daily levels, while accounting for stable, trait-like trajectories over time. In ALT, parameters representing trait-like stability and state-like change are estimated within a single general model. Thus, the ALT framework provides a robust way to statistically model emotion–cognition interactions of interest.

Our ALT analytic strategy was as follows. First, we averaged momentary ratings of positive affect and cognition into a mean daily level for each day in the study assessment periods. Because we were interested in the effects of MBCT on positive affect and cognition, in the final ALT model we focused on days in the post-assessment period following the treatment or waitlist control conditions, controlling for average daily levels of positive affect and cognition in pre-assessment period. Next, a succession of nested univariate ALT models were fitted separately for the repeated positive cognition and the repeated positive affect measures as a means of assessing the temporal dynamics of stability and change within each construct. Each univariate model contained a latent *intercept factor* and latent *slope factor*. The intercept factor was defined such that the factor loadings for all repeated measures were fixed to 1, save for the first assessment of positive cognition or positive affect for each respective study period, which was allowed to covary with the underlying factor. This type of parameterization, called a “predetermined model,” prevents parameter bias from being introduced by not accounting for prior, unassessed levels of the construct (Bollen and Curran, 2004). The intercept factor can be interpreted to index the trait-like aspects of positive cognition and positive affect. Because the trajectory in each construct might vary randomly between individuals over time, we estimated a *linear slope factor* with factor loadings set to 1, 2, 3, 4, and 5 to account for this change.

Next, we estimated autoregressive parameters between daily measures in the presence of the latent growth factors described above. The measure of the average level of positive affect and positive cognition on a given day was regressed upon the measure of that construct on the prior day. We constrained the autoregressive parameters to equality when such constraints did not produce significant decrements in model fit.

Then, the final univariate ALT models were combined into a single multivariate ALT model to examine how positive affect and cognition interact with one another along the upward spiral over the post-assessment period. To account for the “upward spiral” hypothesis (Garland et al., 2010) whereby positive affective and cognitive processes in one point in time may preserve and energize positive affective and cognitive processes in the next, this multivariate model estimated the same autoregressive parameters from the univariate models while testing cross-lagged pathways between the previous day’s level of positive affect on the following day’s level of positive cognition, and vice versa. The multivariate model was then regressed on treatment condition (MBCT vs. waitlist control) and pre-treatment average daily level of positive affect and cognition to evaluate group differences in the pattern of stability and change over time in these measures. Lastly, to test whether MBCT leads to a stronger coupling between positivity on one day and positivity on subsequent days, a two-group multivariate model was then fitted, with parameters estimated separately for the MBCT and waitlist control groups and formally tested for equivalence.

Amos Version 19.0 (Arbuckle, 2010) was used to estimate all ALT models. Of the 130 participants randomly assigned to MBCT (*n* = 64) or the waitlist control condition (*n* = 66), 121 to 129 of these individuals had complete data for any given measure. To handle missing data, the models under investigation employed maximum likelihood estimation procedures, which use all available information from partially missing cases in analyses. Data were missing at random (MAR); the pattern of missingness on positive affect and positive cognition ESM responses varied randomly between individuals, and missingness could not be predicted by the present values on these variables. Only ≤5% of cases were missing data on the analyzed variables. Model fit was evaluated based on the relative chi-square ratio (chi-square/degrees of freedom), using an established rule of thumb of a ratio of less than 3 as indicating acceptable fit (Carmines and McIver, 1981; Marsh and Hocevar, 1985). Model fit was also evaluated using the incremental fit index (IFI; Bollen, 1990), the comparative fit index (CFI; Bentler, 1990), and the root mean squared error of approximation with 90% confidence intervals (RMSEA; Browne and Cudeck, 1993). In addition to the chi-square ratio, adequate model fit was indicated by IFI and CFI values exceeding 0.90 and RMSEA values between 0.08 and 0.10. Because studies have demonstrated that RMSEA values can be significantly inflated in modest sample sizes (∼100 cases; Bollen and Ting, 2000), we expected RMSEA to be overestimated due to sample size. As such, less emphasis is placed on this fit statistic as an accurate index of model fit.

## RESULTS

### UNIVARIATE STATISTICS

**Table 1** displays mean, standard deviation, skewness, kurtosis, and correlation for the 6 days of mean positive affect and cognition in the post-assessment period. Descriptive statistics indicate that, on the whole, participants had a moderate degree of positive affect and cognition each day. Generally, correlations ranged from the small to large range (e.g., 0.3 to 0.8) with most correlations medium in strength. Correlations of mean positive affect across days tended to be high, suggesting stability in participants’ affect, whereas positive cognition ratings were considerably less stable. Same day ratings of positive affect and cognition had the strongest associations. Importantly, all univariate measures of kurtosis and skewness were below 1.0; hence, the use of normal theory maximum likelihood estimation was warranted.

**Table 1:** Correlations and univariate statistics of 6 days of positive affect and positive cognition following MBCT or a waitlist control condition.

	Positive affect						Positive cognition					
	Day 1	Day 2	Day 3	Day 4	Day 5	Day 6	Day 1	Day 2	Day 3	Day 4	Day 5	Day 6
Positive affect day 1												
Positive affect day 2	0.65***											
Positive affect day 3	0.58***	0.65***										
Positive affect day 4	0.54***	0.57***	0.71***									
Positive affect day 5	0.61***	0.61***	0.70***	0.77***								
Positive affect day 6	0.61***	0.60***	0.62***	0.56***	0.66***							
Positive cognition day 1	0.70***	0.36***	0.33***	0.27**	0.38***	0.41***						
Positive cognition day 2	0.37***	0.74***	0.45***	0.45***	0.38***	0.40***	0.35***					
Positive cognition day 3	0.31**	0.37***	0.74***	0.49***	0.42***	0.44***	0.28**	0.42***				
Positive cognition day 4	0.30**	0.34***	0.45***	0.77***	0.50***	0.31***	0.33***	0.44***	0.47***			
Positive cognition day 5	0.45***	0.49***	0.41***	0.56***	0.79***	0.50***	0.42***	0.44***	0.31***	0.52***		
Positive cognition day 6	0.32***	0.30**	0.30**	0.26**	0.34***	0.72***	0.44***	0.41***	0.43***	0.30**	0.41***	
Mean	3.45	3.29	3.25	3.36	3.33	3.27	3.37	3.25	3.28	3.39	3.38	3.31
Standard deviation	1.07	1.08	1.13	1.18	1.17	1.07	0.90	0.92	0.98	1.01	0.99	1.02
Kurtosis	–0.21	–0.10	–0.06	–0.33	–0.76	–0.35	0.24	0.13	0.57	–0.34	–0.22	0.46
Skew	–0.01	0.07	0.13	–0.01	–0.04	–0.10	–0.31	–0.28	–0.26	0.09	–0.08	–0.40

Statistics are presented in aggregate for participants in both the MBCT and waitlist control groups. **p < 0.01; ***p < 0.001.

### EFFECTS OF MBCT ON MOMENTARY POSITIVE COGNITION AND AFFECT

Consistent with our primary hypothesis, multilevel linear regression revealed that compared to the control condition, MBCT was associated with significant increases in momentary positive cognition, *b* = 0.23, SE = 0.07, 95% CI (0.09, 0.36), *p* = 0.001 (see **Table 2**). MBCT participants experienced a significant mean increase in positive cognition from pre- (*M* = 3.96, SD = 0.56) to post-assessment (*M* = 4.21, SD = 0.63), whereas control participants levels of pre- (*M* = 3.92, SD = 0.61) and post-assessment positive cognition (*M* = 3.94, SD = 0.62) were not significantly different. The AR correlation was also significant (*p* < 0.001). Similarly, as reported in the parent study from which the present data is derived (Geschwind et al., 2011), multilevel linear regression revealed that MBCT participants experienced a significant mean increase in positive affect from pre- to post-assessment, whereas control participants did not experience increased positive affect (Geschwind et al., 2011).

**Table 2:** Multilevel regression analysis of the effects of MBCT on momentary experiences of positive cognition.

	Fixed effects		
	*B* (SE)	*t*	*p*
Time (pre vs. post)	0.25 (0.05)	4.97	<0.001
Condition (MBCT vs. WL)	0.28 (0.10)	2.71	0.008
Condition X Time	0.23 (0.07)	3.21	0.001

	**Random effects**		
	***B* (SE)**	***z***	***p***
Intercept	0.25 (0.04)	6.87	<0.001

### POST-ASSESSMENT POSITIVE AFFECT

The post-assessment univariate ALT model for daily positive cognition with an intercept factor and autoregressive structure exhibited excellent fit with the observed data: χ^2^ = 4.98, *df* = 8, *p* = 0.76; CFI = 1.00; IFI = 1.00; RMSEA = 0.00 (0.00, 0.07). Before interpreting parameter estimates, we considered several alternative models. First, we estimated an additional latent slope factor. We assessed for this change because it is possible that any potential effects of treatment on positive affect might strengthen or wane systematically over time. Factor loadings from this slope factor to average daily level of positive affect were fixed to values of 1, 2, 3, 4, 5 on the repeated measures for Days 2 through 6. Adding this latent positive affect slope factor did not improve model fit, nor was the mean of this latent slope factor statistically significant, suggesting that after accounting for the intercept factor and the autoregressive parameters, there was not a systematic trend in changes in positive affect over the 6 days following treatment. Equality constraints were then imposed upon the autoregressive parameters, which did lead to a marginally significant decrement in model fit, χ^2^ *change =* 9.59, *df* = 4, *p* = 0.05. Yet, the constrained model continued to fit the data well: χ^2^ = 14.57, *df* = 12, *p* = 0.27; CFI = 0.99; IFI = 0.99; RMSEA = 0.04 (CI = 0.00, 0.10). The significant variance of the latent intercept factor (*p* < 0.001) indicated that individuals did randomly vary in trait-like level of positive affect over the post-assessment period. All but one of the autoregressive parameters (i.e., between Day 5 and Day 6) were positive and significant in the unconstrained model, and in the constrained model, all the autoregressive effects were positive and significant. These findings indicate that elevations (or decreases) in positive affect on a given day predict elevations (or decreases) in positive affect on the following day.

### POST-ASSESSMENT POSITIVE COGNITION

Similar to the modeling strategy described above, a set of nested models were estimated for positive cognition in the week following treatment. We estimated a post-assessment univariate ALT model for positive cognition with an intercept factor and autoregressive structure; this model fit the data well: χ^2^ = 11.68, *df* = 8, *p* = 0.17; CFI = 0.98; IFI = 0.98; RMSEA = 0.06 (0.00, 0.13). As in the positive affect ALT models, prior to interpreting the parameter estimates, we considered several alternative models. First, we estimated an additional latent slope factor. We assessed for this change because it is possible that any potential effects of treatment on positive cognition might strengthen or wane systematically over time. Factor loadings from this latent slope factor to average daily level of positive cognition were fixed to values of 1, 2, 3, 4, 5 on the repeated measures for Days 2 through 6. As before, adding this latent positive cognition slope factor did not improve model fit, nor was the mean of this latent slope factor statistically significant, suggesting that there was no systematic trend in fluctuations in positive cognition over the post-assessment study period, after accounting for the intercept factor and the autoregressive parameters. Finally, equality constraints were imposed upon the autoregressive parameters, which did not lead to a significant decrement in model fit, χ^2^ *change =* 4.45, *df* = 4, *p* = 0.35. This model fit the data well: χ^2^ = 16.13, *df* = 12, *p* = 0.19; CFI = 0.98; IFI = 0.98; RMSEA = 0.05 (CI = 0.00, 0.11). The significant variance of the latent intercept factor (*p* < 0.001) indicated that individuals did randomly vary in trait-like level of positive cognition over the post-assessment period. However, contrary to our hypothesis, the autoregressive parameters were non-significant, indicating that the propensity toward experiencing positive cognition on the previous day did not influence the tendency to experience positive cognition on the following day. Thus, the observed covariance and mean structure in daily measures of positive cognition was explained sufficiently by a single, underlying, trait-like latent factor whose influence did not systematically vary over time during the post-assessment period.

### INTERRELATION BETWEEN POSITIVE AFFECT AND COGNITION FOLLOWING TREATMENT

A single multivariate ALT model was constructed from the final univariate models of positive affect and cognition. Following Rodebaugh et al. (2002), because we had hypothesized an autoregressive effect in both processes *a priori*, we retained the autoregressive parameters in the multivariate model despite the fact that they were non-significant in the univariate ALT model for positive cognition. In our initial model, we estimated covariances among the two latent intercept factors and the Day 1 measures of positive affect and cognition. This multivariate model had adequate fit: χ^2^ = 109.19, *df* = 45, *p* < 0.001; CFI = 0.95; IFI = 0.95; RMSEA = 0.10 (CI = 0.08, 0.13). Next, we added cross-lagged effects to the model such that Day 2 positive cognition was regressed on Day 1 positive affect, and Day 2 positive affect was regressed on Day 1 positive cognition, etc. This cross-legged pattern was estimated across all 6 days. In addition, correlations were modeled between Day 1 measures of positive affect and positive cognition. Adding these lagged effects to the ALT model resulted in significantly improved model fit, χ^2^ *change =* 22.55, *df* = 10, *p* = 0.013. Next, we imposed equality constraints between the autoregressive parameters for positive affect and positive cognition separately, as well as on the cross-lagged effects. None of these equality constraints diminished model fit. Thus, the final model contained constraints on both the autoregressive and cross-lagged parameters. This model fit the observed data well: χ^2^ = 97.55, *df* = 51, *p* < 0.001; CFI = 0.96; IFI = 0.96; RMSEA = 0.08 (CI = 0.06, 0.11).

Inspection of the final multivariate ALT model revealed a significant autoregressive effect between successive daily measures of positive affect, such that elevations (or decreases) in positive affect on a given day was partially a function of elevations (or decreases) in positive affect on the previous day. Although no such autoregressive effect was observed for positive cognition, the presence of significant cross-lagged effects indicated that elevations (or decreases) in positive affect on a given day influenced elevations (or decreases) in positive cognition on the following day. Furthermore, on each day, there were significant residual covariances for daily positive affect and positive cognition (*r*’s ranging from 0.75 to 0.82). Taken together, the multivariate ALT model results indicated that daily positive affect was driven by a stable, trait-like component and level of positive affect from the preceding day, whereas positive cognition was driven by a stable trait-like component as well as the previous day’s level of positive affect.

### THE INFLUENCE OF MBCT

Going beyond the unconditional multivariate ALT model described above, we sought to evaluate the influence of mindfulness training on the pattern of stability and change in positive affect and cognition by regressing the final multivariate model on a single, dichotomous item representing treatment condition (MBCT vs. waitlist control) and the average daily level of positive affect and cognition prior during the pre-assessment period. The unconstrained model fit the data fairly well: χ^2^ = 185.53, *df* = 69, *p* < 0.001; CFI = 0.92; IFI = 0.92; RMSEA = 0.11 (CI = 0.09, 0.13). We identified a significant effect of treatment condition on the latent intercept factor of positive affect (*p* < 0.05) such that MBCT participants had significantly higher trait-like positive affect in the post-assessment period than participants in the waitlist control condition. However, controlling for effects of treatment on the latent intercept of positive affect, the effect of treatment condition on the latent intercept of positive cognition was non-significant (*p* = 0.11). In addition, average pre-assessment levels of positive affect and cognition were significantly associated with the latent intercept of post-assessment positive affect (*p* < 0.001) and cognition (*p* < 0.001), respectively.

**FIGURE 2:**
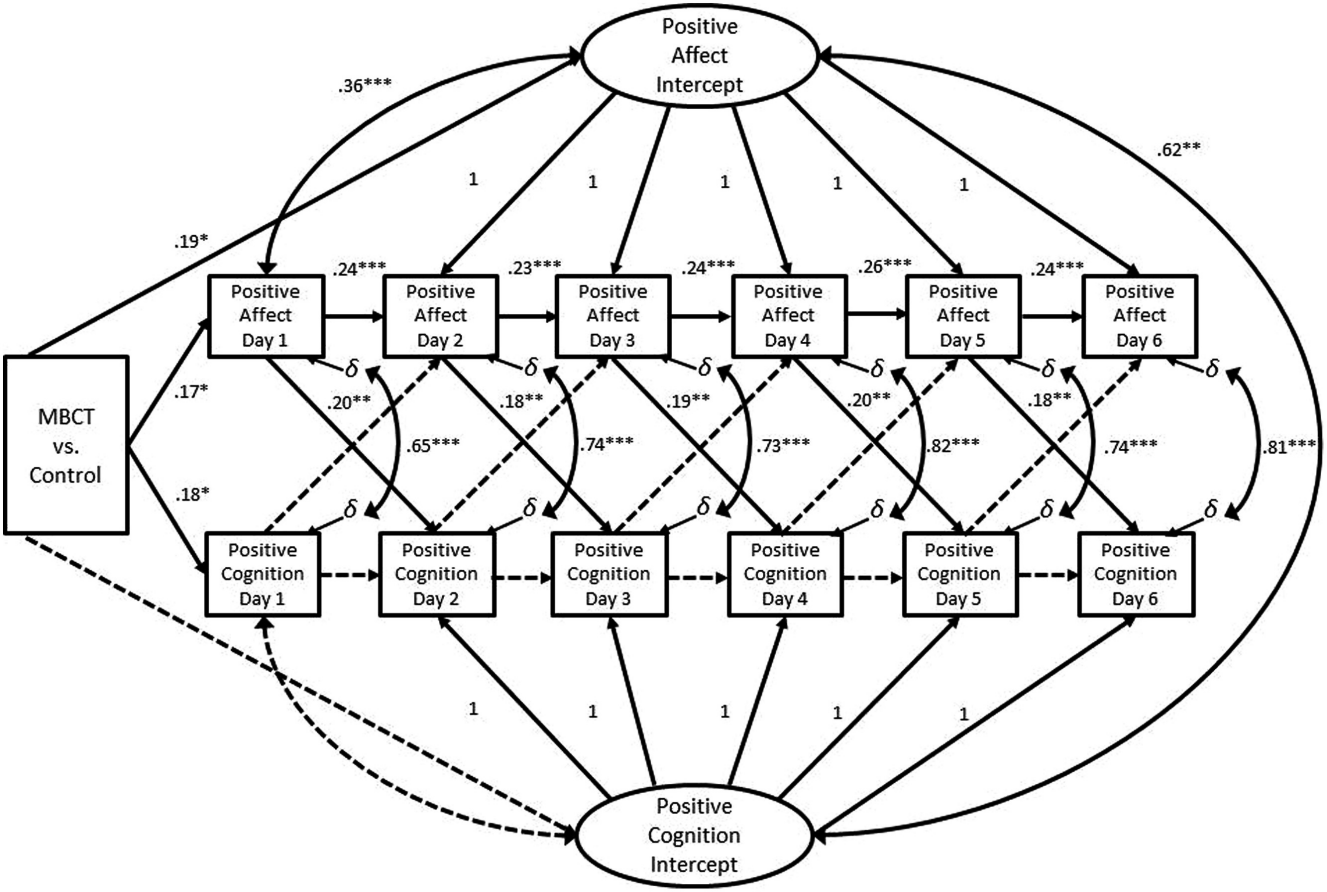
**Final autoregressive latent trajectory model of the influence of mindfulness training on upward spirals of positive emotion and cognition.** This model contains post-assessment data from both the MBCT and control group; the influence of treatment condition on daily positive affect and cognition is modeled with a dichotomous predictor variable. Note – only statistically significant factor loadings and parameter estimates are shown. Although not depicted for the sake of clarity, latent positive affect and cognition factors were regressed onto average daily level of positive affect and cognition during the 6-day pre-assessment period. Similarly, error terms and non-significant parameter estimates are not shown. Non-significant paths are represented by dashed arrows with no parameter estimates. Correlations are represented by double-headed arrows. Factor loadings are marked with the value of 1; all other parameter estimates are standardized regression weights. *p ≤ 0.05; **p < 0.01; ***p < 0.001.

Next, we imposed equality constraints between the autoregressive parameters for positive affect and positive cognition separately, as well as on the cross-lagged effects. None of these equality constraints significantly diminished model fit. Thus, the final model contained constraints on both the autoregressive and cross-lagged parameters. This model fit the observed data adequately: χ^2^ = 197.08, *df* = 85, *p* < 0.001; CFI = 0.92; IFI = 0.92; RMSEA = 0.10 (CI = 0.08, 0.12). Restraining the model did not lead to a significant decrement in model fit, χ^2^ *change =* 11.62, *df* = 16, *p* = 0.77. In the constrained model, treatment condition remained a significant predictor of the latent intercept of positive affect (*p* = 0.03). As in the final unconditioned multivariate ALT model, we identified significant autoregressive effects on state-like positive affect, significant cross-lagged effects between positive affect and the following day’s level of positive affect and cognition, and significant within-day covariances between positive affect and cognition. Significant parameter estimates for this final model are presented in **Figure 2**. These findings suggest that participants who experienced higher levels of positive affect and cognition following an 8-weeks course of MBCT were more likely to continue to experience positive emotions and thoughts energized by the momentum of a cycle of self-reinforcing positive affect.

Lastly, we fitted a series of two-group multivariate models, with autoregressive and cross-lagged parameters estimated separately for the MBCT and waitlist control groups, controlling for the average daily level of positive affect and cognition prior during the pre-assessment period. We first allowed autoregressive and cross-lagged parameters to vary freely. Next, we imposed equality constraints between the autoregressive parameters for positive affect and positive cognition separately, as well as on the cross-lagged effects. None of these equality constraints significantly diminished model fit. In the final model, autoregressive and cross-lagged parameters were constrained. This final model the data well: χ^2^ = 256.40, *df* = 144, *p* < 0.001; CFI = 0.92; IFI = 0.92; RMSEA = 0.07 (CI = 0.06, 0.09). Significant autoregressive effects of positive affect were observed for both the MBCT group (*b* = 0.26, SE = 0.09, *p* = 0.004) and the waitlist control group (*b* = 0.22, SE = 0.08, *p* = 0.009), but there were no significant autoregressive effects for positive cognition in either group. Significant cross-lagged effects of positive affect on the following day’s level positive cognition were observed for the MBCT group (*b* = 0.17, SE = 0.08, *p* = 0.04), but there were no significant cross-lagged effects in the waitlist control group (*b* = 0.11, SE = 0.07, *p* = 0.10). Significant residual covariances were observed for same day positive affect and positive cognition for both groups. The latent intercept factors of positive affect and positive cognition significantly covaried in MBCT group (*b* = 0.22, SE = 0.08, *p* = 0.004) but not in the waitlist control group (*b* = 0.07, SE = 0.04, *p* = 0.10). In sum, with regard to model equivalence, models for the MBCT and waitlist control groups differed on: (a) cross-lagged effects of positive affect on positive cognition; and (b) covariance of latent positive affect and latent positive cognition intercept factors.

## DISCUSSION

The current study revealed the temporal dynamics and interactivity of positive affect and positive cognition following treatment of partially remitted depression with MBCT. Study findings supported some of our key hypotheses: MBCT enhanced moment-by-moment positive cognition from pre- to post-treatment, and elevated daily levels of positive affect predicted higher levels of positive affect and positive cognition experienced on following days. Moreover, MBCT appeared to strengthen the cross-lagged relationship between current positive affect and positive cognition on the following day. However, contrary to our hypotheses, current positive cognition was not a significant predictor of later positive cognition or positive affect on succeeding days. Taken together, results demonstrate that at least over a 6 days period, daily positive affect and positive cognition are energized by prior experiences of positive affect – providing partial support for our hypothetical upward spiral model. Mindfulness training may play an important role in this emotionally driven upward spiral by virtue of its apparent ability to stimulate positive affect and cognition among persons with deficits in positive affectivity.

In ALT models, both positive affect and cognition were found to exhibit trait-like properties, suggesting that a significant portion of the variance in each factor as it is expressed from day to day is attributable to an underlying tendency toward experiencing positive emotions and pleasant thoughts, respectively. Yet, study findings also suggest the presence of state-like, time-specific effects whereby positive affect and cognition interact and maintain one another over time. Thus, results showed that a classical state-trait model (without a latent slope or growth structure) was sufficient to represent positive affect and cognition. Study findings suggest that positive affect is the prime mover in the upward spiral, whereby prior levels of positive affect govern subsequent experiences of positive affect and cognition. This notion is congruent with central tenets of the broaden-and-build theory, which suggest that, far from being epiphenomena, positive emotions exert consequential effects on cognition that over time build durable psychosocial resources. A number of mechanisms may account for the effects of positive affect on cognition, including: the broadening of the scope of attention (Fredrickson and Branigan, 2005; Rowe et al., 2007); the tuning of attentional selection toward affect-congruent stimuli (Friedman and Förster, 2010); the activation of state-dependent memories (Blaney, 1986); the promotion of positive evaluations of others (Forgas and Bower, 1987); the induction of positive interpretational biases (Mathews, 2012); or the increased dopaminergic activity in projections from the ventral striatum to the prefrontal and anterior cingulate cortices (Ashby et al., 1999; Mitchell and Phillips, 2007) which may serve as the neural substrate of the aforementioned processes. Through these and other putative mechanisms, affective state may color or even control the valence and quality of cognition (Clore and Palmer, 2009). The significant cross-lagged effects of daily positive affect on subsequent affect and cognition, as well as the high correlations between positive cognition and same day positive affect, suggest that emotion and thought are tightly coupled in the form of a resonant loop (Isen et al., 1978) that may preserve itself in an autoregressive fashion over time.

Although participants assigned to both the MBCT and waitlist control conditions exhibited significant autoregressive effects on positive affect, positive affect and cognition were more tightly coupled among MBCT participants following mindfulness training, as evidenced by the presence of significant cross-lagged effects within the MBCT group that were absent in the control group. This data provides partial support for our hypothesis that mindfulness training may spark an upward spiral of positive psychological processes. Findings indicate that for those individuals who respond to MBCT by experiencing enhanced positive affect, these enhancements tend to maintain themselves through a self-reinforcing cycle that is largely impelled by emotion. Thus, effects from the initial post-assessment boost in positive affect and cognition produced by mindfulness training may reverberate through subsequent iterations of the emotion–cognition cycle, thereby preserving and energizing a partial upward spiral dynamic as time progresses. Yet, to be clear, the presence of a full upward spiral could not be established, as no cross-lagged effects from positive cognition to positive affect were observed.

While mindfulness training may enhance positive affect, it may have consequential effects on negative affect as well. Indeed, in this same study sample, MBCT was found to significantly reduce momentary experiences of negative affect, and these decreases, along with increases in momentary positive affect, statistically mediated the therapeutic effect of MBCT on depressive symptoms (Batink et al., 2013). These findings converge with a meta-analysis of controlled trials which found mindfulness-based interventions to significantly reduce negative affective states (Goyal et al., 2014). At the same time, mindful emotion regulation is thought to involve increased sensory awareness of emotional experience, as evidenced by neuroimaging studies indicating a reduction in midline prefrontal reactivity in support of enhanced limbic (e.g., insular) circuitry activation (Farb et al., 2012). Thus, far from suppressing or avoiding negative emotional states, mindfulness is associated with increased negative emotion differentiation that predicts consequent reductions in negative emotional lability (Hill and Updegraff, 2012). These other affective features of mindfulness are also important, in that negative emotions can confer insights that are crucial for longer-term psychological health and development (Parrott, 2014). Integrating findings on mindfulness and negative affect with data from the current study, it is possible that rather than merely eliminating negative emotions, mindfulness training may engender therapeutic effects by increasing the ratio of positive-to-negative emotions (Fredrickson, 2013). Although the exact mathematical parameters of this ratio remain unknown, it seems that when negative emotions are balanced by contextually appropriate positive emotions, this affective balance may decouple negative affective states from maladaptive psychological outcomes.

Beyond previously established effects of mindfulness on positive affect (Geschwind et al., 2011), to our knowledge current study findings are the first in the literature to indicate that treatment with MBCT is associated with significant increases in momentary positive cognitions (i.e., positive thought appraisal). Mindfulness is typically held to be a non-discursive, non-conceptual psychological process that is putatively antithetical to cognitive appraisal (Chambers et al., 2009). In contrast to this assumption, results of the present investigation indicate that partially remitted depressed participants of a MBCT course experienced significantly more positive momentary appraisals of their thoughts following mindfulness training. These findings provide partial support for Garland et al. (2009, 2011) hypothesis that mindfulness training promotes positive (re)appraisals by allowing for decentering from current negative situational appraisals into a non-conceptual, broadened metacognitive state from which an enlarged scope of previously unattended contextual information can be accessed for the generation of new, positive appraisals of self and world. Clearly, mindfulness and positive cognition are not identical psychological processes; to the contrary, mindfulness practice is commonly held to evoke states where sensory experience is monitored without any cognitive elaboration or evaluation (Hölzel et al., 2011). Yet, we argue that positive affect and cognition may be adaptive byproducts of mindfulness practice. According to our theoretical model (Garland and Fredrickson, 2013), the positive affect induced by mindfulness practice may provide a signal which tunes the attentional system to detect stimuli that are congruent with the induced emotional state, resulting in greater awareness of the pleasant, beautiful, or rewarding aspects of one’s own mental experience or life circumstances. While mindfulness may temporarily suspend evaluative language during decentering from negative automatic thoughts, individuals will inevitably re-engage their socially constructed autobiographical narratives to make meaning out of their lives (Olivares, 2010). As the result of positive emotional tuning of the information processing system afforded by mindfulness practice, when individuals return to the semantic-narrative mode from the state of mindfulness, the new appraisals and thoughts emerging from conscious reflection on life circumstances or spontaneous insight may tend to have a positive valence. Through these processes, mindfulness training may promote positive reappraisal and facilitate traditional cognitive restructuring techniques in CBT.

The facilitative effects of mindfulness meditation training on positive emotion–cognition interactions in persons with a history of depression may have consequential effects on physical health. Indeed, meditation-induced upward spirals of positive emotion have been found to promote vagally driven increases in heart rate variability that are mediated by increased perceptions of social connectivity (Kok et al., 2013). Similarly, mindfulness training has been shown to enhance savoring of natural reward as evidenced by cue-elicited increases in heart rate variability and decreased heart rate (Garland et al., 2014b). Thus, inducing positive emotions through meditative practices seems to enhance positive appraisals of socioenvironmental stimulus contexts, a cognitive process that then exerts downstream salutary effects on cardiac-autonomic function. Such effects may indeed be consequential for health; in that regard, depression severity has been inversely associated with attenuated heart rate variability (Kemp et al., 2010), and patients exhibiting major depressive disorder with melancholia have attenuated heart rate variability relative to healthy controls which may have adverse effects on mortality and morbidity (Kemp et al., 2014). More studies are needed to elucidate the impact of meditation-induced upward spirals on vagal function, affective tone, and longevity itself.

Strengths of the present study include: the use of ESM to measure changes in positive psychological processes; the use of multiple measurement points to assess the process of stability and change; and the use of ALT modeling, a statistical technique that confers numerous advantages with regard to its ability to estimate causal models and flexibly estimate inter- and intra-individual variation over time. In addition, the study was strengthened by its randomized controlled design, which minimized potential internal threats to validity.

The study also had a number of limitations. First, because no active control condition was employed, we are unable to determine if the effects of MBCT on positive affect and cognition were specifically due to mindfulness training, or due to non-specific therapeutic factors (e.g., attention by a caring professional, social support, group dynamic, expectation of benefit, etc.). It is possible that other treatments (e.g., CBT) might have similar facilitative effects on positive psychological processes following treatment. In addition, we lacked objective treatment fidelity measures to determine to what extent instructors adhered to the MBCT protocol. The ESM procedure was also limited in that participants self-reported their completion time for ESM reports; thus, our ability to conclusively determine whether a given experience sampling report was valid or invalid (i.e., late) was somewhat reduced; however, earlier research demonstrates that self-reported completion times are reliably accurate (Jacobs et al., 2005). Also, ESM positive affect scores were derived from principal components analysis; however, some scholars assert that a latent variable factor analytic approach produces more valid data (Preacher and MacCallum, 2003). Further, our sample was comprised of persons with a history of major depressive disorder; thus, study conclusions may not generalize to non-depressed samples. Lastly, the upward spiral model includes attentional mechanisms which were not measured in the present study. Future studies employing an active control group, as well as a broader array of self-report measures, behavioral tasks (e.g., a dot probe task comparing attentional responses to pleasant vs. neutral stimuli), and psychophysiological methods [e.g., analysis of heart rate variability or the late positive potential (LPP) component of the electroencephalogram] would allow for a more robust exploration of how MBCT might impact cognition–emotion interactions and a more complete test of the upward spiral model.

In conclusion, the present study suggests that positive affect and positive cognition are driven by upward spiral processes that might potentially be stimulated through mindfulness training. As such, this work adds to the growing body of research indicating that positive psychological processes have direct clinical relevance for the treatment of persons suffering from emotion dysregulation (Garland et al., 2010). Future studies are needed to elucidate how self-reinforcing cycles of positive psychological experience may be sparked by metacognitive processes, enhanced cognitive flexibility, and the selective tuning of attentional and interpretational systems to apprehend the positively valenced features of the natural and human environment.

## Conflict of Interest Statement

The authors declare that the research was conducted in the absence of any commercial or financial relationships that could be construed as a potential conflict of interest.
